# Characteristics of Peripheral Retinal Refraction and Its Role in Children with Different Refractive States

**DOI:** 10.1155/2024/7712516

**Published:** 2024-08-08

**Authors:** Qi Zhao, Yanhua Wang, Tiangang Liang, Weixiang Nie, Pei Xue, Jie Cheng

**Affiliations:** ^1^ Shanxi Aier Eye Hospital Aier Eye Hospital Group, Changsha, China; ^2^ Aier Eye Hospital Jinan University, Guangzhou, China

## Abstract

**Introduction:**

Peripheral retinal refraction plays a crucial role in myopia, but the specific mechanism is not clear. We refined the retinal partitions to explore the characteristics of peripheral retinal refraction and its role in emmetropic, low, and moderate myopic children aged 6 to 12 years.

**Methods:**

A total of 814 subjects (814 eyes) were enrolled in the study. The participants were divided into three groups according to the central spherical equivalent refraction (SER), which were emmetropia group (E), low myopia group (LM) and moderate myopia group (MM). Multispectral refractive topography (MRT) was used to measure the retinal absolute and relative refractive difference value (RDV) in different regions. The range was divided into superior, inferior, temporal, and nasal RDV (SRDV, IRDV, TRDV, and NRDV) on the basis of several concentric circles extending outward from the macular fovea (RDV15, RDV30, RDV45, RDV30–15, RDV45–30, and RDV-45). Kruskal–Wallis test was used to analyze the differences of peripheral refraction for all the regions among the three groups. Spearman rank correlation was performed to explore correlations between SER and RDV, axial length (AL) and RDV.

**Results:**

The absolute value of RDV decreased with increasing degree of myopia in all regions (*P* < 0.01). Subjects with different refractive degrees had different relative value of RDV. In nasal position within 45° and temporal position within 30°, the peripheral retina exhibited significantly different relative hyperopic refractive status among Group *E*, Group LM, and Group MM (*P* < 0.05). SER was negatively correlated with NRDV within 30° (especially in the range of NRDV30–15) (*r* = −0.141, *P* < 0.01), positively correlated with TRDV within 15° (*r* = 0.080, *P* = 0.023), and not significantly correlated with SRDV and IRDV when the retina was divided into four parts. AL was positively correlated with NRDV within 30° (especially in the range of NRDV30–15) (*r* = 0.109, *P* = 0.002), negatively correlated with TRDV within 15° (*r* = −0.095, *P* = 0.007).

**Conclusions:**

The peripheral defocus has significant implications for the genesis of myopia. The peripheral defocus of the horizontal direction, especially within the range of NRDV30, has greater effect on the development of myopia in children. Higher NRDV30 is associated with lower SER and longer AL.

## 1. Introduction

Myopia is a leading cause of visual impairment and has raised significant international concern in recent decades with rapidly increasing prevalence and incidence worldwide. It is estimated that by 2050 there will be almost 50% of the world population with myopia and almost 10% with high myopia [1]. The fact is that the incidence and progression rates of myopia and high myopia are high in Chinese schoolchildren [2]. Prevention and control of myopia has become a crucial public health priority in China.

Current animal and human research has demonstrated that myopia development was a result of the interplay between genetic and environmental factors [3, 4], but the specific mechanism that explained myopia has been elusive. In recent years, it has been proved that peripheral defocus has important influence on the growth of eyes. Specially, peripheral hyperopia defocus is a high-risk factor for the occurrence and progression of myopia [5]. Eyes with emmetropia and hyperopia have a relative peripheral myopic refractive state, while eyes with myopia have a relative peripheral hyperopic refractive state [6]. Previous studies [7, 8] have investigated the relative refractive status of different regions of the peripheral retina. Researchers either simply studied the retinal relative difference value (RDV) at several concentric areas, or investigated the RDV at four sectors only, including superior, inferior, nasal, and temporal RDV. In our study, we combined these partitioning methods, that is, we divided the retina into four regions on the basis of concentric circles. Then, we measured RDV in different regions to further explore the effect of peripheral retinal refractive status on myopia.

With the revival of modern technology, great strides have been made in the development and application of medical inspection device. Multispectral refractive topography (MRT) is a novel instrument using multispectral imaging technology, which can comprehensively analyze the peripheral refraction of the regional retina. The advent of MRT provides convenience for our peripheral defocus-related research. This study aimed to investigate the peripheral refractive status in children with different refractive states and its role in the occurrence and development of myopia.

## 2. Participants and Methods

This was a descriptive cross-sectional study. It was approved by the Ethics Committee of Shanxi Aier Eye Hospital (EYESX-20220303-01) and adhered to the tenets of Declaration of Helsinki. And informed consent was obtained from all subjects. From July 2021 to April 2022, a total of 814 subjects (814 eyes) who were admitted to the Pediatric Ophthalmology Department of Shanxi Aier Eye Hospital were enrolled in the study. The inclusion criteria were as follows: (1) subjects age 6 to 12 years, (2) subjects have refractive errors ranging from +0.50 D to −6.00 D, astigmatism of less than or equal to −2.00 D, (3) subjects with a best corrected visual acuity of 20/20 or better, and (4) subjects who can cooperate to complete the eye examination. The exclusion criteria were as follows: (1) subjects with intraocular pressure higher than 21 mmHg, (2) subjects with obvious strabismus (exotropia ≥ 15Δ, esotropia ≥ 10Δ), (3) subjects with other corneal diseases, such as keratoconus, corneal scarring, corneal trauma, et al., (4) familial genetic diseases and systemic diseases affecting eye health, (5) a history of previous ocular surgery that may influence refraction, and (6) a history of myopia therapy like corneal contact lens, defocus incorporated multiple segments spectacle lenses, atropine usage, and low-level red-light therapy, et al.

All participants underwent a comprehensive examination, including slit-lamp examination, detection of strabismus, intraocular pressure (IOP), cycloplegic subjective refraction, axial length (AL), and MRT examination. Cycloplegic subjective refraction was achieved after four successive tropicamide eye drops instilled 5 minutes apart. The participants were divided into three groups according to the central spherical equivalent refraction (SER), which were emmetropia group (E, ≤+0.50 D and ≥−0.50 D), low myopia group (LM, <−0.50 D and ≥−3.00 D), and moderate myopia group (MM, <−3.00 D and ≥−6.00 D).

**Figure 1:**
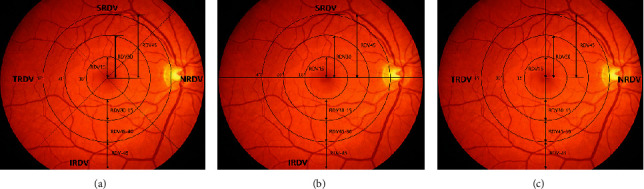
Three different partition method. (a) Three concentric areas with 15-degree intervals from fovea (RDV15, RDV30, RDV45, RDV30–15, RDV45–30, and RDV-45) and four sectors (SRDV, IRDV, NRDV, and TRDV). (b) Three concentric areas with 15-degree intervals from fovea (RDV15, RDV30, RDV45, RDV30–15, RDV45–30, and RDV-45), and two sectors (SRDV and IRDV). (c) Three concentric areas with 15-degree intervals from fovea (RDV15, RDV30, RDV45, RDV30–15, RDV45–30, and RDV-45), and two sectors (NRDV and TRDV).

MRT was used to measure the retinal absolute and relative RDV in different regions after mydriasis. Retinal absolute RDV is the specific refraction at a certain place on the retina. Retinal relative RDV is the refractive difference between a place on the retina and the macular fovea. The ranges included several areas extending outward from the macular fovea (RDV15, RDV30, RDV45, RDV30–15, RDV45–30, and RDV-45), and superior RDV (SRDV), inferior RDV (IRDV), temporal RDV (TRDV), and nasal RDV (NRDV) (Figure 1). The results of refractive measurement were DS/DC × *θ* (DS = diopter sphere, DC = diopter cylinder, and *θ* = astigmatism axis). The SER was calculated using the formula DS + DC/2.

IBM SPSS software (version 26.0) was used for the statistical analysis. GraphPad Prism software (version 8.4) was used to draw statistical charts. Kruskal–Wallis test was used to analyze the differences of absolute and relative peripheral refraction for all the ranges among the three groups. A Spearman rank correlation was performed to explore correlations between SER and RDV, AL and RDV. *P* < 0.05 was considered statistically significant.

## 3. Results

All participants aged from 6 to 12 years with a male to female ratio of 397 : 417. Best corrected visual acuity was 20/20 in all participants. The general data of the three groups are shown in Table 1.

### 3.1. Comparison Results of RDV among the Three Groups Using the Partition Method in Figure 1(a)

**Figure 2:**
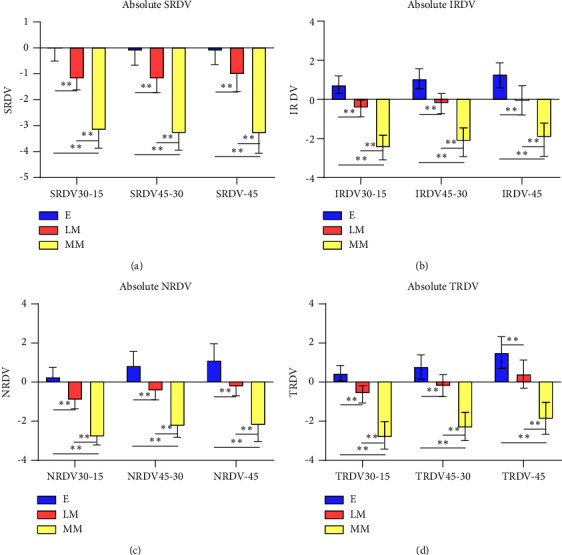
The absolute RDV of different peripheral retinal ranges using the first partition method (Figure 1(a)) in three groups. (a–d) represent the RDV of the superior, inferior, nasal, and temporal sides, respectively. ^*∗*^*P* < 0.05, ^*∗∗*^*P* < 0.01.

**Figure 3:**
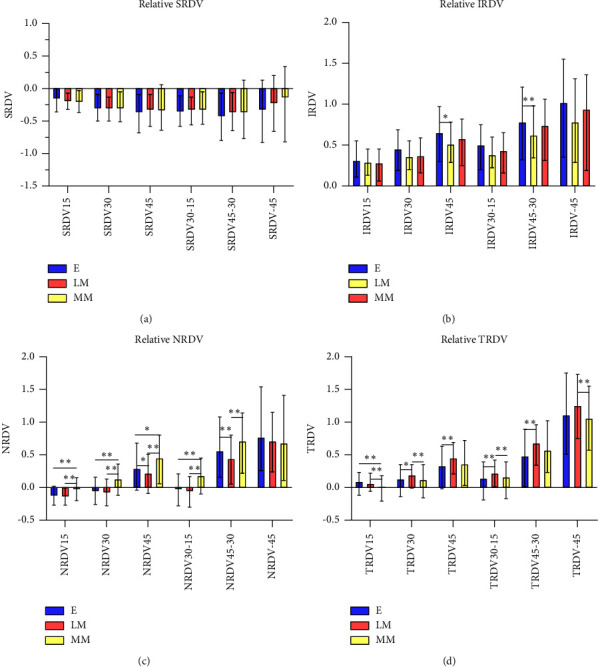
The relative RDV of different peripheral retinal ranges using the first partition method (Figure 1(a)) in three groups. (a–d) represent the RDV of the superior, inferior, nasal, and temporal sides, respectively. ^*∗*^*P* < 0.05, ^*∗∗*^*P* < 0.01.

All four regions of the absolute peripheral retinal refraction RDV, including SRDV, IRDV, NRDV, and TRDV, showed the lowest value of RDV in the moderate myopia group (*P* < 0.01) on the whole (Figure 2). And three groups showed an increasing trend in the value of RDV of four regions from retinal center to periphery. The peripheral retina of all groups presented relative myopia in the superior position, while presented relative hyperopia in the inferior position, and there was no significant difference in the relative RDV among the three groups (Figure 3). It presented relative hyperopia in temporal position and nasal area beyond 30° of the fovea. In nasal position within 45°, the peripheral retina of Group LM exhibited a lower relative hyperopic defocus than Group *E* (*P* < 0.05) and Group MM (*P* < 0.01). In temporal position within 30°, the peripheral retina of Group LM exhibited a slightly higher relative hyperopic refractive status than Group *E* (*P* < 0.05) and Group MM (*P* < 0.01).

The Spearman rank correlation analysis indicated a negative correlation of SER with NRDV15, NRDV30, NRDV45, and NRDV30–15 (*P* < 0.05); it indicated a positive correlation of SER with TRDV15 (*P* < 0.05). Besides, there was no significant correlation between SER and RDV in other ranges (Table 2).

### 3.2. Comparison Results of RDV among the Three Groups Using the Partition Method in Figure 1(b)

**Figure 4:**
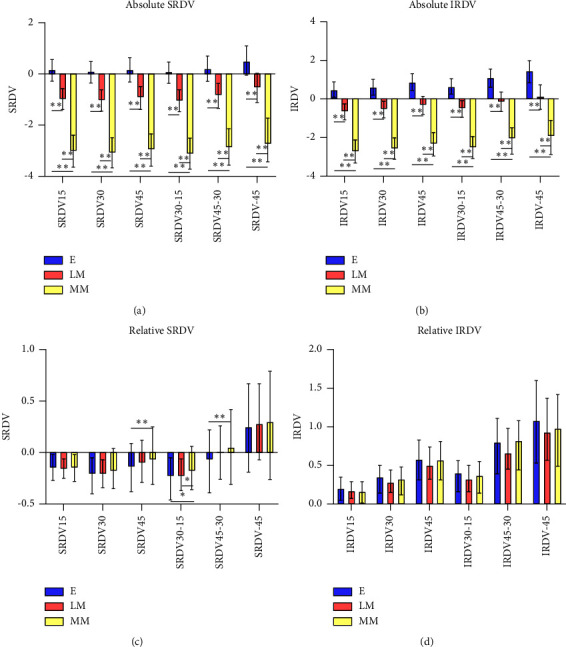
(a, b) The absolute RDV of different peripheral retinal ranges using the second partition method (Figure 1(b)) in three groups. (a) and (b) represent the RDV of the superior and inferior sides, respectively. ^*∗*^*P* < 0.05, ^*∗∗*^*P* < 0.01. (c, d) The relative RDV of different peripheral retinal ranges using the second partition method (Figure 1(b)) in three groups. (c) and (d) represent the RDV of the superior and inferior sides, respectively. ^*∗*^*P* < 0.05, ^*∗∗*^*P* < 0.01.

The absolute value of SRDV and IRDV decreased with increasing degree of myopia (*P* < 0.01) (Figures 4(a) and 4(b)). And three groups showed an increasing trend in the value of RDV of the two regions from retinal center to periphery. In the superior position, the refractive status of all three groups presented a shift from relative myopic defocus to relative hyperopic defocus, with a statistically significant difference in the value of RDV between the Group MM and the Group *E* in the range of RDV30–15 (*P*=0.018), RDV45–30 (*P*=0.006), and RDV45 (*P*=0.008). In the inferior position, the peripheral retina of the three groups had a relative hyperopic refractive status. And the value of RDV increased, but there was no statistically significant difference among the three groups (Figures 4(c) and 4(d)).

The Spearman rank correlation analysis indicated a negative correlation of SER with SRDV45 and SRDV45–30 (*P* < 0.05); it indicated a positive correlation of SER with IRDV45 (*P*=0.035). Besides, there was no significant correlation between SER and RDV in other ranges (Table 3).

### 3.3. Comparison Results of RDV among the Three Groups Using the Partition Method in Figure 1(c)

**Figure 5:**
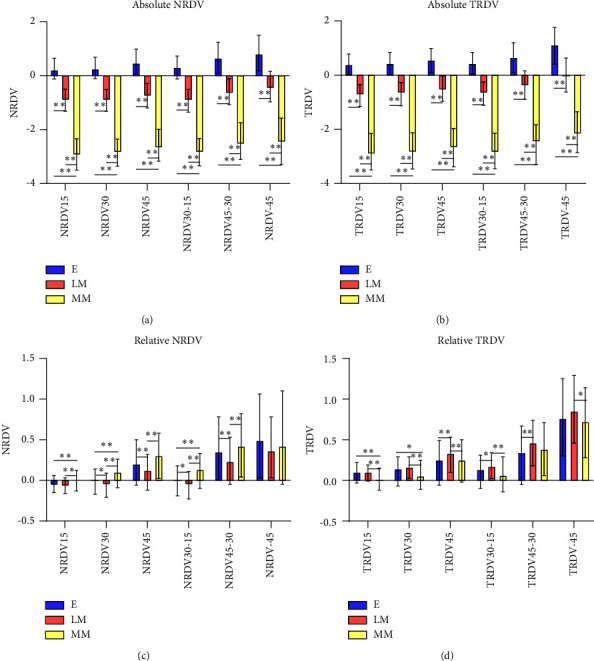
(a, b) The absolute RDV of different peripheral retinal ranges using the third partition method (Figure 1(c)) in three groups. (a) and (b) represent the RDV of the nasal and temporal sides, respectively. ^*∗*^*P* < 0.05, ^*∗∗*^*P* < 0.01. (c, d) The relative RDV of different peripheral retinal ranges using the third partition method (Figure 1(c)) in three groups. (c) and (d) represent the RDV of the nasal and temporal sides, respectively. ^*∗*^*P* < 0.05, ^*∗∗*^*P* < 0.01.

The absolute value of NRDV and TRDV decreased with increasing degree of myopia (*P* < 0.01) (Figures 5(a) and 5(b)). And three groups showed an increasing trend in the value of RDV of the two regions from retinal center to periphery. In the nasal position, the value of RDV increased from center to periphery. Within 30° area, the peripheral retina of Group MM exhibited higher relative hyperopic refractive status than other two groups (*P* < 0.05). And within 45°area, Group LM had a lower relative hyperopic defocus than Group MM (*P* < 0.01). In temporal position within 30°, the peripheral retina of Group LM exhibited a higher relative hyperopic refractive status than Group MM (*P* < 0.01) (Figures 5(c) and 5(d)).

The Spearman rank correlation analysis indicated a negative correlation of SER with NRDV30 and NRDV30–15 (*P* < 0.01); it indicated a positive correlation of SER with TRDV15 and TRDV30 (*P* < 0.05). Besides, there was no significant correlation between SER and RDV in other ranges (Table 4).

### 3.4. Correlation of AL with Relative Value of RDV at Nasal and Temporal Retina Dividing According to the Partition Method in Figure 1(a)

The results indicated there was a positive correlation between AL and relative value of NRDV15, NRDV 30, and NRDV30–15 (*P* < 0.01), while there was a negative correlation between AL and relative value of TRDV15 (*P* < 0.01) (Table 5).

## 4. Discussion

Myopia is a complex disease caused by many risk factors. At present, there is still no theoretical system that can explain the cause and development of myopia, the mechanism has certain limitations. Various relevant research studies have shown that the main theories related to the pathogenesis of myopia in recent years included accommodative lag theory [9, 10], peripheral defocus theory, neurotransmitter-related theory [11–13], and scleral hypoxia theory [14, 15]. More attention has been paid to the relationship between peripheral defocus and myopia. Retinal peripheral hyperopic defocus was first recognized as a risk factor for myopia in the 1970s [16]. Researchers found that pilots who had retinal relative hyperopic state were more likely to suffer from myopia. Since then, experiments in animal models have provided convincing evidence that peripheral retinal hyperopic defocus could produce dramatic increases in axial growth and myopia even in presence of a clear fovea image [17–19]. In recent years, researchers have indicated myopic eyes exhibit relative peripheral hyperopia [20, 21] and high myopic eyes had higher degree of relative peripheral hyperopia [7].

This study explored the characteristics of absolute and relative peripheral refraction in emmetropic, low myopic, and moderate myopic children aged 6 to 12 years old. Our results showed that the absolute or relative refractive states of peripheral retinal different regions were different in children with emmetropia, low myopia, and moderate myopia. When the retina was divided into four parts, superior regions exhibited relative peripheral myopia, while inferior regions exhibited relative peripheral hyperopia. Whether the retina was divided into four parts (i.e., superior, inferior, nasal, and temporal regions) or two parts (i.e., superior and inferior regions or nasal and temporal regions), the difference of peripheral hyperopia defocus at horizontal meridian was more significant than that at vertical meridian. Therefore, we inferred that compared with the vertical direction of the retina, the peripheral defocus of the horizontal direction had a greater effect on the development of myopia in children. Xiaoli et al. [7] compared the horizontal and vertical retinal relative peripheral refraction in the children with low myopia. They speculated that there may be imbalance between vertical and horizontal eye development during the development of myopia. Similarly, Atchison et al. [22] demonstrated that, for adults aged 18–35, myopia had more effect on peripheral refraction along the horizontal than along the vertical visual field. These researches were consistent with the results of the present study. The findings of Ho et al. suggested human retina was able to differentiate defocused signals of different regions [23]. And differences in response to spherical defocus between different retinal regions could potentially provide cues for controlling eye growth [24]. However, the specific mechanism and reasons for it need to be further explored.

In the present study, we found that nasal RDV within 30 was the most associated with the development of myopia, among all the factors. Spearman correlation analysis showed that SER was negatively correlated with nasal RDV within 30°, positively correlated with temporal RDV within 15°, and not significantly correlated with vertical RDV (SRDV and IRDV) when the retina was divided into four parts. Previous studies have indicated a strong linear relationship between SER and AL. Myopia is mainly due to the excessive elongation of the AL [25, 26]. The result was further supported by the correlation between AL and RDV. AL was positively correlated with nasal RDV within 30° and negatively correlated with temporal RDV within 15°. We thought that peripheral defocus may affect AL and then develop myopia. Research suggested that the sensitivity and distribution of neurons and cells of retina may lead to different retinal responses to peripheral defocus in different regions, which led to different effects on AL of the eyes [27–29]. Further investigation was necessary to figure out the specific mechanism. When RDV in the horizontal direction was explored alone, the results similarly indicated a strong negative correlation between SER and RDV in the NRDV30 and a strong positive correlation between SER and TRDV15. When only the superior and inferior regions were divided, SER was found to be negatively correlated with SRDV45–30 and positively correlated with RDV beyond the inferior 45°. We speculated it may be caused by the inclusion of part of the nasal and temporal regions in this division method. Xiaoli et al. [7] conducted a study on 90 cases of Chinese children aged 5–18, and discovered peripheral refraction of RDV within the range of 45−30 may be closely related to the development of myopia. Zheng et al. [8] demonstrated that the eccentricities between 20 and 35° RDV may be closely related to refractive development and eye growth in young aged 18–28 years. These differences were mostly because their division was different from that of this study.

Based on the principle of peripheral defocus, several optical treatment methods have emerged in clinical practice, such as orthokeratology, multifocal contact lenses, peripheral defocus spectacle lenses, and defocus incorporated multiple segments spectacle lenses. These methods can induce retinal peripheral myopia defocus (or reduce retinal peripheral hyperopia defocus) to achieve the effect of myopia control [30]. Autorefractometers are currently the gold standard for testing refraction of the central retina. Liao et al. [31] proved that autorefractometry and MRT show high agreement in measuring central refraction, and MRT could provide a potential objective method to assess peripheral refraction. Hence, the advantages of MRT can be used to solve more problems for myopia in the future.

Above all, in the development of myopia, the peripheral defocus has significant implications for the genesis of myopia. The peripheral defocus of the horizontal direction has a greater effect on the development of myopia in children. Moreover, RDV within the range of NRDV30 is most closely associated with the development of myopia. Nevertheless, a limitation of the present study is the narrow range of samples selected. Further research with a larger sample size is needed to be conduct in the future.

## Figures and Tables

**Table 1:** The general data of participants in each group.

Parameter	Group E	Group LM	Group MM
Age (years)	8 (7, 9)	8 (7, 9)	10 (8, 11)
Gender (M : F)	90 : 119	223 : 207	84 : 91
DS (D)	0 (−0.25, +0.25)	−1.50 (−2.00, −1.00)	−3.50 (−4.00, −3.25)
DC (D)	−0.50 (−0.50, 0)	−0.50 (−0.50, 0)	−0.50 (−1.00, 0)
SER (D)	−0.25 (−0.44, 0)	−1.63 (−2.25, −1.25)	−3.75 (−4.38, −3.50)
IOP (mmHg)	15.3 ± 2.9	15.5 ± 2.9	15.3 ± 2.9

DS: diopter sphere; DC: diopter cylinder; SER: spherical equivalent refraction; IOP: intraocular pressure.

**Table 2:** Correlation analysis results between SER and RDV in different ranges.

Range	*r*	*P*
SRDV15	0.065	0.063
SRDV30	0.011	0.752
SRDV45	−0.020	0.564
SRDV30–15	−0.001	0.970
SRDV45–30	−0.034	0.327
SRDV-45	−0.052	0.141
NRDV15	−0.124	≤0.001^*∗∗*^
NRDV30	−0.140	≤0.001^*∗∗*^
NRDV45	−0.077	0.027^*∗*^
NRDV30–15	−0.141	≤0.001^*∗∗*^
NRDV45–30	−0.049	0.163
NRDV-45	0.038	0.275
IRDV15	0.054	0.121
IRDV30	0.069	0.050
IRDV45	0.059	0.092
IRDV30–15	0.068	0.051
IRDV45–30	0.045	0.196
IRDV-45	0.048	0.168
TRDV15	0.080	0.023^*∗*^
TRDV30	0.017	0.635
TRDV45	−0.025	0.470
TRDV30–15	0.000	0.996
TRDV45–30	−0.044	0.209
TRDV-45	0.056	0.113

^*∗*^ *P* < 0.05, ^*∗∗*^*P* < 0.01.

**Table 3:** Correlation analysis results between SER and RDV in different ranges.

Range	*r*	*P*
SRDV15	0.037	0.294
SRDV30	−0.043	0.224
SRDV45	−0.072	0.039^*∗*^
SRDV30–15	−0.060	0.087
SRDV45–30	−0.080	0.022^*∗*^
SRDV-45	−0.015	0.674
IRDV15	0.042	0.226
IRDV30	0.026	0.460
IRDV45	0.025	0.482
IRDV30–15	0.020	0.576
IRDV45–30	0.022	0.525
IRDV-45	0.074	0.035^*∗*^

^*∗*^ *P* < 0.05.

**Table 4:** Correlation analysis results between SER and RDV in different ranges.

Range	*r*	*P*
NRDV15	−0.061	0.080
NRDV30	−0.100	0.004^*∗∗*^
NRDV45	−0.049	0.159
NRDV30–15	−0.106	0.003^*∗∗*^
NRDV45–30	−0.027	0.437
NRDV-45	0.018	0.615
TRDV15	0.139	≤0.001^*∗∗*^
TRDV30	0.077	0.028^*∗*^
TRDV45	0.010	0.771
TRDV30–15	0.059	0.090
TRDV45–30	−0.020	0.560
TRDV-45	0.048	0.170

^*∗*^ *P* < 0.05, ^*∗∗*^*P* < 0.01.

**Table 5:** Correlation analysis results between AL and relative value of RDV at nasal and temporal retina.

	NRDV15	NRDV30	NRDV45	NRDV30–15	NRDV45–30	NRDV-45
*r*	0.109	0.110	0.050	0.109	0.025	−0.029
*P*	0.002^*∗∗*^	0.002^*∗∗*^	0.150	0.002^*∗∗*^	0.482	0.409

	TRDV15	TRDV30	TRDV45	TRDV30–15	TRDV45–30	TRDV-45

*r*	−0.095	−0.041	0.011	−0.026	0.031	−0.029
*P*	0.007^*∗∗*^	0.242	0.764	0.455	0.380	0.409

^*∗∗*^ *P* < 0.05.

## Data Availability

The datasets used and/or analyzed during the current study are available from the corresponding author on reasonable request.
